# Recent Progress in Plasmonic Biosensing Schemes for Virus Detection

**DOI:** 10.3390/s20174745

**Published:** 2020-08-22

**Authors:** Elba Mauriz

**Affiliations:** 1 Department of Nursing and Physiotherapy, Universidad de León, Campus de Vegazana, 24071 León, Spain; elba.mauriz@unileon.es; 2 Institute of Food Science and Technology (ICTAL), La Serna 58, 24007 León, Spain

**Keywords:** plasmonics, virus detection, nanomaterials, SPR, LSPR, SERS, colorimetric, electrochemiluminescence

## Abstract

The global burden of coronavirus disease 2019 (COVID-19) to public health and global economy has stressed the need for rapid and simple diagnostic methods. From this perspective, plasmonic-based biosensing can manage the threat of infectious diseases by providing timely virus monitoring. In recent years, many plasmonics’ platforms have embraced the challenge of offering on-site strategies to complement traditional diagnostic methods relying on the polymerase chain reaction (PCR) and enzyme-linked immunosorbent assays (ELISA). This review compiled recent progress on the development of novel plasmonic sensing schemes for the effective control of virus-related diseases. A special focus was set on the utilization of plasmonic nanostructures in combination with other detection formats involving colorimetric, fluorescence, luminescence, or Raman scattering enhancement. The quantification of different viruses (e.g., hepatitis virus, influenza virus, norovirus, dengue virus, Ebola virus, Zika virus) with particular attention to Severe Acute Respiratory Syndrome Coronavirus 2 (SARS-CoV-2) was reviewed from the perspective of the biomarker and the biological receptor immobilized on the sensor chip. Technological limitations including selectivity, stability, and monitoring in biological matrices were also reviewed for different plasmonic-sensing approaches.

## 1. Introduction

Viral infections can be transmitted among human populations in a short period of time via droplets, aerosols, contact with abiotic surfaces, and even fecal matter [1,2,3]. The serious consequences of rapid airborne transmission are a cause of great concern since the global pandemic of coronavirus disease (COVID-19) has surpassed the number of cases and economic impact of recent virus disease outbreaks [e.g., H1N1/H5N1 flu, Ebola, MERS-CoV (Middle East Respiratory Syndrome Coronavirus), and SARS-CoV-1(Severe Acute Respiratory Syndrome Coronavirus 1)] [4]. The need for effective management of current and future epidemics has urged the development of rapid and sensitive screening tools. Therefore, viral diagnosis has become a primary need to control the spread of viral diseases [4,5,6,7,8,9].

Traditionally, viral diagnosis has relied upon culture-based methods and serological tests that detect either direct (intact viruses or their components: proteins or nucleic acids) or indirect (immunogens or antibodies against the viral antigens) virus infectious diseases [5].

More recently, the advances of molecular techniques in viral diagnosis have permitted that nucleic acid-based amplification methods such as the polymerase chain reaction (PCR) and the reverse transcriptase-quantitative polymerase chain reaction (RT-qPCR) have consolidated as the gold standard methods for the routine diagnosis of a wide range of viruses (human immunodeficiency virus (HIV), hepatitis B and C viruses, and cytomegalovirus (CMV)) [10,11,12,13]. In spite of the inherent advantages of PCR and RT-qPCR tests regarding sensitivity and selectivity, the utilization of time-consuming protocols (the report of results usually takes 1–3 days) and the high false-negative rate, limit their effectiveness to prevent the risk of new infections [4].

Consequently, the requirement for fast and cost-effective viral diagnostic methods has led to putting the focus on the development of real-time biosensing platforms. Among the variety of biosensor technologies that have arisen in the last decades for virus detection, plasmonic applications have gained significant attention, owing to their versatility, label-free monitoring, and low time of response [8,10,14]. The potential for multiplexing and system miniaturization are additional benefits for the point-of-care testing [15]. The combination of these features with the possibility of exploiting the physical and electronic properties of nanomaterials has allowed the design of ultrasensitive detection formats. In this way, novel plasmonic configurations taking advantage of colorimetric, surface-enhanced Raman scattering (SERS), and electrochemiluminescence sensing schemes seem to be ideally suited for monitoring either intact viruses or their components [16,17]. From this perspective, the fabrication of nanopatterned structures has emerged as a key factor for viral diagnosis using arrays of nanoplasmonic antennas [18]. The improvement in the spatial resolution of plasmonic substrates along with the enhancement of surface-to-volume ratios makes possible the detection of single virus particles with higher sensitivities [19].

On the other hand, the chemical activation of the surface plays a major role for improving the sensing efficiency of single virus particles [20,21]. The biofunctionalization of chemically activated substrates using aptamers, antibodies, antigens, or nucleic acids may enable the selective targeting of virus. Likewise, the design of effective surface coverages ensures the adequate orientation of biological receptors and, thereby, facilitate direct capture of intact virus from complex biological media.

Thus, nanoplasmonic biosensors provide a promising approach to attain ultra-low detection limits of viral particles, antigens, or nucleic acids from clinical specimens (i.e., blood, serum, saliva, etc.). The vast majority of virus sensing plasmonic applications are based on the well-known operation principles of surface plasmon resonance (SPR) biosensors. Nonetheless, the success in achieving optimal biosensing performance requires the design of novel biosensing strategies capable of maintaining the specificity and sensitivity of measurements while preserving the biocompatibility of the immobilized biological receptor.

Therefore, the aim of this review was to present recent advances in plasmonic biosensing schemes for the detection of virus on the basis of signal enhancement. This review specially focused on the utilization of sensing surfaces comprising nanomaterials-based approaches. Current progress in the development of plasmonic-based applications involving the combination with colorimetric, electrochemical, fluorescence, or fiber-optic detection principles is also discussed.

## 2. Biosensing Strategies

**Figure 1:**
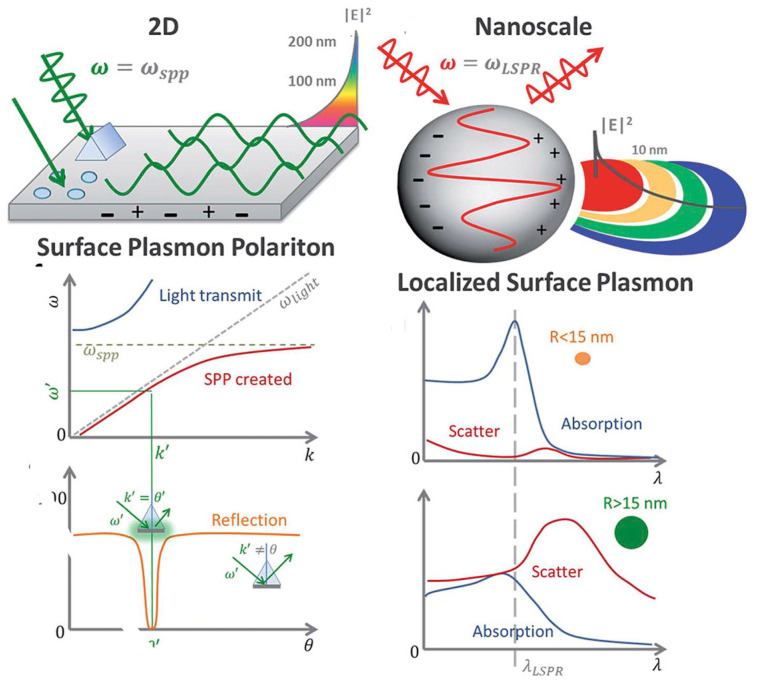
Differences in volume, surface, and localized surface plasmon resonances of zero-dimensional metallic nanoparticles and two-dimensional thin, metallic surfaces. The surface plasmon polariton (SPP) can only be excited at certain wave vectors and decays evanescently from the surface. The momentum-matching condition leads to the SPP resonance and only exists at certain incident angles. In localized surface plasmon resonance, absorption dominates for small particles, less than ~15 nm, whereas the scattering cross-section dominates for big nanoparticles, greater than ~15 nm. Adapted with permission from Li et al. [26] Copyright © (2015) Royal Society of Chemistry (RSC)).

The fundamental principles of plasmonic biosensors rely on the propagation of surface plasmons along the interface of a thin, metal layer (commonly noble metal such as gold) and a dielectric (aqueous medium). A detailed description of the optical working principles was comprehensively reviewed in previous works and it is beyond the scope of this review [22,23,24]. In short, plasmonic biosensing takes advantage of the local refractive index changes of the transducer surface when monitoring molecular interactions between the target analyte and the immobilized biological receptor [9,17,22,25]. Binding events occurring in the surface can be monitored in two distinct forms: SPR and localized surface plasmon resonance (LSPR). Both SPR and LSPR depend on the refractive index of the surrounding media to induce spectral shifts. However, the dimension of the plasmonic nanomaterial determines the difference between SPR (metallic, thin layers) and LSPR [26], being below the wavelength of incident light in the latter (Figure 1).

**Figure 2:**
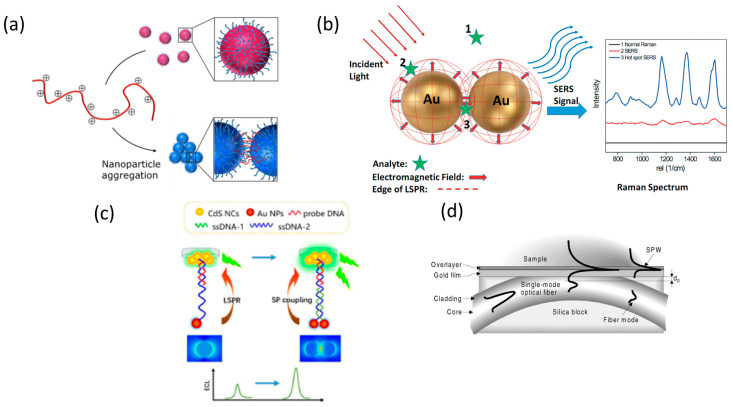
Schematic representation of plasmonic configurations: (**a**) Colorimetric assay showing dispersed (top) and aggregated (bottom) nanoparticles. Adapted with permission from Gormley et al. [28] Copyright © (2014) American Chemical Society (ASC). (**b**) Surface-enhanced Raman spectroscopy (SERS) phenomenon for an organic analyte on gold nanoparticles (AuNPs). Adapted with permission from Wei et al. [30] Copyright © (2015) RSC. (**c**) Schematic illustration of plasmonic-enhanced electrochemiluminescence (ECL) for DNA hybridization. Adapted with permission from Li et al. [31] Copyright © (2016) RSC. (**d**) Surface Plasmon Resonance (SPR) sensing structure based on a side-polished, single-mode fiber. Adapted with permission from Slavík et al. [33] Copyright © (2001) Elsevier.

This special characteristic allows enhancing the spatial resolution of LSPR configurations by designing the geometry and composition of metallic nanostructures, laying the basis for colorimetric plasmonic biosensing [27,28]. The local electromagnetic field can enhance optical processes such as Raman scattering and fluorescence, leading to SERS and plasmon-enhanced electrochemiluminescence (ECL) sensing schemes [29,30,31]. The sensitivity achieved by both optical configurations is higher than that of SPR and LSPR, thus ensuring the detection of single virus particles. Alternatively, the propagation of electromagnetic radiation using optical fibers can also be applied to virus detection via miniaturized platforms with SPR and LSPR configurations [32,33] (Figure 2).

Technological advancements in plasmonic biosensing including colorimetric and fluorescence enhancement as well as the utilization of nanomaterials and optical aperture nanostructures for achieving highly sensitive virus detection are described in this section.

### 2.1. Plasmonic Nanomaterials

Nanomaterials provide the signal amplification of plasmonic biosensing due to their interesting optical, magnetic, and electrical properties. The biocompatibility and easy chemical activation are also significant advantages. Likewise, the large surface area-to-volume ratio provides a higher loading of biological receptors [14,34,35,36]. Depending on their chemical composition, dimension, and physical properties, common types of plasmonic nanomaterials can vary from metallic nanoparticles and quantum dots to graphene nanostructures (i.e., carbon nanocomposites: Nanotubes, nanosheets, nanoflowers) [14,37,38,39]. Since the number of nanomaterial-based applications for viral sensing has increased significantly in the last decade, a comprehensive review of recent developments on plasmonic platforms is presented below and summarized in Table 1.

#### Metal Nanoparticles

Plasmonic nanomaterials can be classified regarding their chemical composition in organic (fullerenes, carbon nanotubes) and inorganic (metal, oxide-based nanomaterials, and quantum dots) materials. Among the latter ones, noble-metal nanoparticles have been extensively applied to plasmonic biosensing due to the strong absorption of light resulting from the oscillation of the free electrons. The size and shape of nanoparticles along with the interparticle distance determine the amplitude of oscillation and the position of the SPR band [57]. Likewise, variations of the chemical surrounding media due to the binding event between the biological receptor and the target analyte can also affect the oscillations. This implies that the physicochemical properties of the metallic nanoparticles can be tuned to transform LSPR spectra and produce even color variations, generating a signal response [19,27]. Additionally, their high biocompatibility makes possible the functionalization of the surface with a broad range of biological receptors from antibodies to nucleic acids, glycoproteins, and aptamers, thus enabling the selective binding of a variety of analytes [35,36]. Therefore, the utilization of gold and silver nanoparticles in LSPR, SERS, fluorescence enhancement, and colorimetric assays represents a popular approach for detecting small analytes.

**Figure 3:**
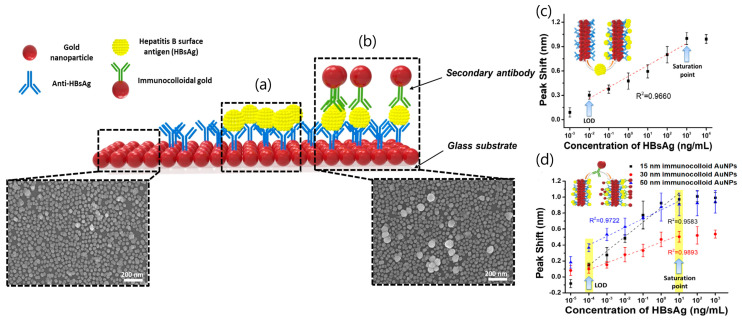
**Localized Surface Plasmon Resonance** (LSPR) biosensing chip and analytical performance: (**a**) Gold nanoparticles arrayed on glass substrate; (**b**) modified hetero-assembled, AuNPs sandwich-immunoassay LSPR chip format; (**c**) detection of hepatitis B surface antigen (HBsAg) by single assay LSPR sensing chip format; and (**d**) modified hetero-assembled, AuNPs sandwich-immunoassay LSPR chip format using immunocolloid AuNPs. Absorbance spectra of both conjugated and unconjugated AuNPs were measured and compared in the wavelength range from 700 to 400 nm. Changes in the spectrum peak at different concentrations of HBsAg (1 pg mL^−1^ to 1 μg mL^−1^ HBsAg) were monitored using 15, 30, and 50 nm of immunocolloid AuNPs as signal enhancers. Adapted with permission from Kim et al. [40] Copyright © (2018) Elsevier.

LSPR sensing is the most common sensing scheme that exploits noble-metal nanoparticles in analytical applications. LPSR biosensors employing gold nanoparticles (AuNPs) have been successfully applied to clinical diagnostics, environmental monitoring, and food safety [23]. Particularly, a LSPR platform consisting of a hetero-assembled, AuNPs sandwich-immunoassay has been reported to detect hepatitis B surface antigen (HBsAg) at 100 fg mL^−1^ [40]. First, a glass substrate fabricated with AuNPs and conjugated with an anti-HBsAg antibody was prepared to detect target antigen HBsAg (Figure 3). After 10 min of inoculation, a second layer of AuNPs conjugated with anti-HBsAg antibody was formed to obtain the hetero-assembled, AuNPs sandwich-immunoassay chip format. The specificity of the immunoassay was tested against alpha fetoprotein (AFP), C-reactive protein (CRP), and prostate-specific antigen (PSA) while the validation was carried out in serum samples. Nevertheless, the suitability of the test for multiplexing analysis is suggested for further research.

**Figure 4:**
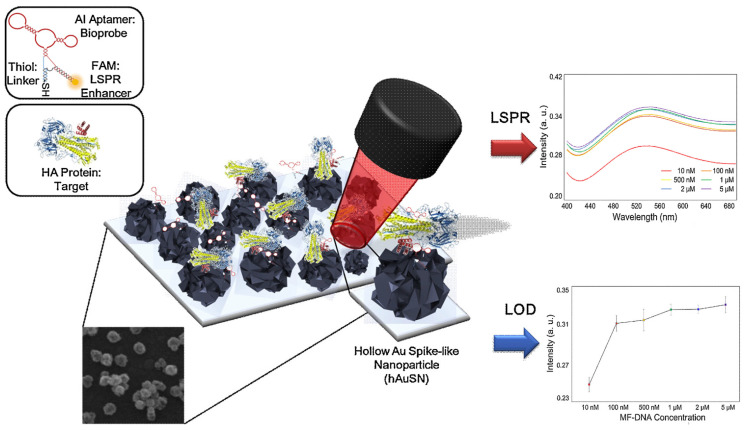
Schematic image of the LSPR detection biosensor for avian influenza virus (AIV H5N1). Absorbance spectra were measured in the 400–700-nm wavelength range. The target–aptamer interaction induced changes in the local refractive at a given wavelength that were attributed to the extension of light absorption by the biofilm on the Au spike-like nanoparticle (hAuSN)-modified indium-tin-oxide (ITO) electrode. The effect of DNA three-way unction (3WJ) concentration on the LSPR substrate was investigated in the concentration range from 10 nM to 5 μM. Error bar represents relative standard deviation of five independent experiments. Adapted with permission from Lee et al. [41] Copyright © (2019) Elsevier.

Another LSPR platform takes advantage of a hollow Au spike-like nanoparticle (hAuSN) to detect avian influenza virus (AIV H5N1) using a multi-functional DNA three-way unction (3WJ) [41]. This method provides target recognition by aptamer binding, connection to the substrate, and signal enhancement. To achieve this multi-functionality, each piece of the DNA 3WJ was tailored to a hemagglutinin (HA) binding aptamer, fluorescein (FAM) dye, and thiol group. The application comprised the immobilization of spike-like gold nanoparticles onto indium-tin-oxide (ITO) substrates and the subsequent functionalization with the DNA for detecting hemagglutinin (HA) protein at 1 pM levels. Although the work focused on the characterization of the hAuSN-modified ITO substrate and the DNA 3WJ immobilization process, the performance of clinical tests as well as the specificity of LSPR measurements with related and nonrelated proteins in 10-diluted chicken serum were also discussed (Figure 4).

The LSPR effect of silver nanostructures has also been reported to detect dengue NS1 antigen using an immunoassay format [42]. The creation of the metal nanostructures was obtained through the deposition of a thin, silver film onto silicon substrate via thermal annealing. The repeated recrystalization of the thin, silver layer resulted in the formation of the nanostructures in which the anti-NS1 antibody was immobilized. To measure NS1 antigen in whole blood, a polyethersulfone membrane filter was integrated at the inlet of the biosensor. This membrane allowed the separation of plasma from whole blood, avoiding the centrifugation of the sample. The direct detection of NS1 antigen was performed with a limit of detection of ~0.06 μg mL^−1^, which is within the clinical levels observed in the first days of dengue infection. Despite the proposed method making use of spiked, whole blood samples instead of patient samples, the blood-plasma separation-integrated strategy represents a great advance toward the development of lab-on-chip devices and point-of-care virus diagnostics.

A singular LSPR immunosensor approach compares the capability of antibody-functionalized gold, silver, and copper nanoparticles for the detection of respiratory syncytial virus (RSV) in the presence of ***Pseudomonas aeruginosa*** and adenovirus [43]. The characterization of nanoparticle size distribution and time-dependent LSPR shifting was determined for both nonfunctionalized and functionalized metallic nanoparticles. The interaction with RSV showed better results in LPSR shifting for copper as compared to gold and silver nanoparticles with a LOD and limit of quantification (LOQ) of 2.4 and 14 plaque-forming units (PFU), respectively. Regarding the cross-reactivity under the influence of other respiratory pathogens, copper and silver nanoparticles were more specific for RSV monitoring. Although additional results on the detection of RSV clinical specimens are not shown, this work presents good preliminary results for the direct determination of RSV.

**Figure 5:**
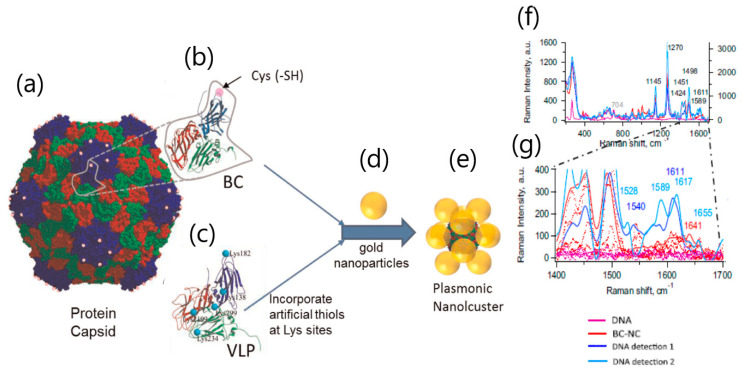
Plasmonic nanoclusters’ synthesis. (**a**) The protein capsid (30 nm in diameter) generated from (**b**) protein subunit where pink circles represent Cys incorporated via genetic engineering for a total of 60 thiols per capsid, basic cluster (BC)-mutant. (**c**) Protein subunit of the virus like particles (VLP) showing the locations of the naturally occurring Lys. (**d**) Gold nanoparticles (24–30 nm in diameter) were bound to the VLP or BC by directed self-assembly resulting in (**e**) plasmonic nanoclusters. (**f**) Detection of DNA by individual BC nanoclusters (NCs), averaged Raman spectra of plain DNA (violet), BC NC (red), and BC NC after addition of DNA (light and dark blue; DNA detections 1 and 2 were done individually in separate clusters). (**g**) Expanded area (1400–1700 cm^−1^) with specific DNA Raman peaks. The characterization of NCs in solution showed a major peak of 78 nm and 77 nm for VLP-NC and BC NC, respectively, thus indicating that the size of the NC is larger relative to the free AuNP (major peak: 37 nm). Adapted with permission from Lebedev et al. [44] Copyright © (2016) Elsevier.

Plasmonic nanoparticles have also been exploited by SERS biosensors. The sensing scheme of SERS benefits from the enhancement of Raman scattering by providing the modulation of surface plasmon polaritons at a greater distance from the metallic nanoparticle surface, thereby offering higher sensitivity than that of SPR and LSPR. Lebedev et al. reported a SERS platform applied to the detection of viral DNA using nanoclusters (NC) of gold nanoparticles [44]. SERS experiments were performed on lone gold nanoparticles, individual nanoclusters, and in two distinct plasmonic nanoclusters. NC were conjugated to either virus-like particles (VLP) incorporating artificial thiols at the Lys sites (VLP-NC) or basic cluster (BC)-mutant incorporating genetic engineering Cys to the protein capsid subunit (Figure 5). This work showed the potential of fabricating complex 3D nanoplasmonic structures in solution by assembling gold nanoparticles on VLP. The sensitivity of the nanocluster biosensor was demonstrated as a proof of principle for the detection of M13 mp18 single-stranded DNA below 0.25 ng μL^−1^ levels.

**Figure 6:**
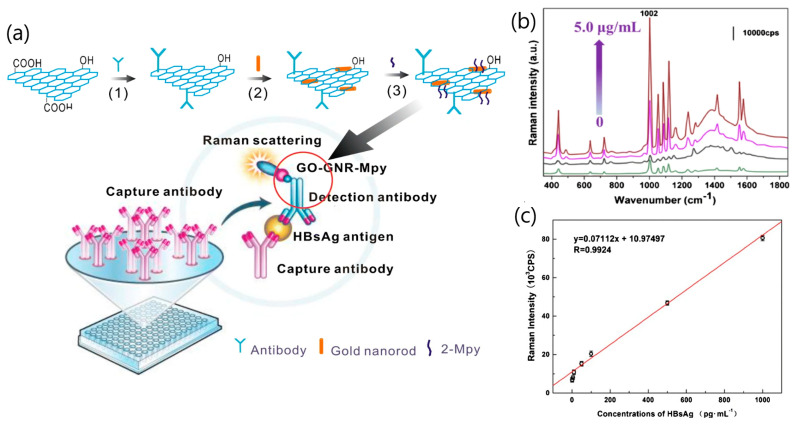
(**a**) Schematic illustration of Raman immunoassay based on graphene oxide gold nanorods (GO-GNRs). (1) GO was functioned by anti-HBsAg antibody. (2) GNRs were decorated on the surface of GO to form GO-GNRs’ composites. (3) The 2-mercaptopyridine (2-Mpy) was bound to the GNRs’ surface by forming the S-Au bond. (**b**) SERS assays of different concentrations of HBsAg based on the GO-GNR Raman immunosensor (the concentrations of HBsAg were 0.01 pg∙mL^−1^ (green), 1.0 pg∙mL^−1^ (black), 10 ng∙mL^−1^ (purple), and 5.0 μg∙mL^−1^ (red); (**c**) the linear relationship of SERS intensity with HBsAg concentrations in the range of 1–1000 pg∙mL^−1^. The accumulation time and laser power were set as 20 seconds and 1.0 mW for each spectrum, respectively. To determine the HBsAg contents more sensitively, the SERS signals of samples were collected with 785 nm Ar^+^ ion laser sources. The data were collected by averaging five measurements. Adapted with permission from Miu et al. [45] Copyright © (2016) Springer.

Another virus SERS-based strategy has been proven to detect hepatitis B surface antigen HBsAg [45]. The biosensor utilized an active tag consisting of a composite of graphene oxide (GO) containing carboxy and hydroxy groups decorated with gold nanorods (GNRs). Each composite carried the SERS probe 2-mercaptopyridine (Mpy) in which the antibody against HBsAg was immobilized (Figure 6a). The immunoassay took advantage of the sandwich format between the capture antibody and the detection antibody attached to the GO-GNRs’ composites by measuring the SERS signal provided via the 2-Mpy bound to the GNRs’ surface (Figure 6b). The SERS signal response to the interaction of the HBs’ Ag was measured in the 1–1000 pg·mL^−1^ with a limit of detection of 0.05 pg mL^−1^ (at a signal to noise ratio of 3). The applicability of the proposed method for clinical analysis was evaluated by testing HBsAg in spiked, real serum samples from nine patients showing recovery values between 96 and 104%.

### 2.2. Other Plasmonic Nanoparticles

#### 2.2.1. Quantum Dots

Heavy metal quantum dots are semiconductor nanomaterials that have gained prominence in recent years for multiplex detection due to their broad adsorption and narrow emission band. The possibility of tuning the emission spectra over a wide range of wavelengths and the resistivity to external physico-chemical conditions are also significant advantages [58]. Among them, fluorescent quantum dots (QD) have unique optical properties, which, in combination with the surface plasmon properties of metallic nanoparticles, can enhance the sensitivity of plasmonic biodetection systems [46,47,48,59]. Typically, most of quantum dots’ applications have been exploited in LSPR-based biosensors because the distance and dimensions of the adjacent gold nanoparticles can affect the fluorescence signal and, therefore, be quenched depending on the analyte concentration. Thus, various interesting LSPR developments have made use of the fluorescence enhancement to detect very low dimensional samples like viruses.

**Figure 7:**
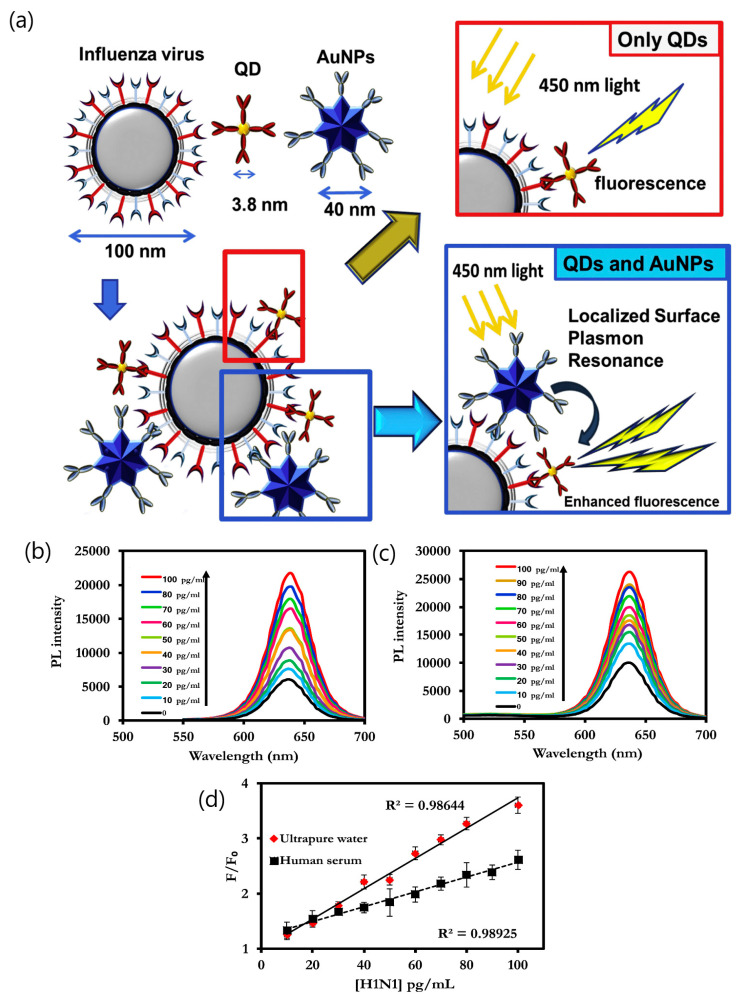
(**a**) Schematic representation of the detection principle for the influenza virus using the LSPR-induced fluorescence nanobiosensor. Photoluminescence emission spectra showing wavelength changes and corresponding photoluminescence calibration curves in (**b**) ultrapure deionized water and (**c**) in human serum. (**d**) Corresponding calibration curve for detection of the influenza virus in deionized (DI) water and in human serum. Errors bars in (**d**) denote standard deviation of three replicate measurements. Adapted with permission from Takemura et al. [46] Copyright © (2017) Elsevier.

**Figure 8:**
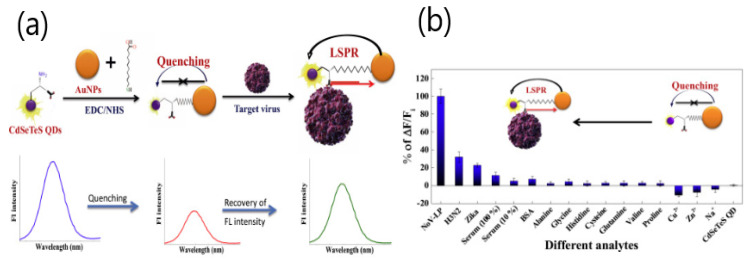
(**a**) Schematic diagram for the preparation of CdSeTeS QD (quantum dot)/AuNPs’ nanocomposites and their sensing mechanism toward norovirus-like particles (NoV-LPs’) detection. The sample solution was excited at 450 nm, and the fluorescence intensity was measured in a range of 500–700 nm. The close covalent attachment of AuNPs with CdSeTeS QDs effectively quenched the fluorescence signal, which was recovered after NoV-LPs’ entrapment. (**b**) Selectivity test of the Ab-CdSeTeS QDs/AuNPs’ nanobiosensor with 30 μg mL^−1^ of influenza, 104 PFU (plaque forming units) mL^−1^ of Zika viruses, and other common amino acids and interfering metal ions. Adapted with permission from Nasrin et al. [48] Copyright © (2018) Elsevier.

Various virus-based applications involving LSPR signal enhancement with fluorescent quantum dots have been reported by Park’s group. For instance, Takemura et al. described a LSPR immunosensor to determine influenza H1N1 virus [46]. The biosensor utilized the fluorescence signal generated by alloyed CdSeTeS QDs functionalized with anti-neuraminidase antibody (anti-NA Ab) in combination with the optical transduction induced from AuNP conjugated to anti-hemagglutinin antibody (anti-HA Ab). The enhancement of the fluorescent signal triggered by the LSPR signal from adjacent AuNPs allowed the sensitive detection of the antigens on the surface of H1N1 virus in deionized water (0.03 pg mL^−1^) and human serum (0.4 pg mL^−1^) (Figure 7). The effect of the distance between fluorescent CdZnSeS/ZnSeS QDs and gold nanoparticles was studied by Nasrin et al. using different lengths of peptide chains as a linkage [47]. The sensing mechanism was enhanced by the quenching of the QDs’ fluorescence due to the steric hindrance on the LSPR signal induced by the different concentrations of the influenza virus. By controlling the distance between QDs and gold nanoparticles with a peptide chain of 18 amino acids, the influenza virus was determined specifically with a detection limit of 17.02 fg mL^−1^. The same quenching effect was observed when using CdSeTeS QDs/AuNPs’ nanocomposites for detecting norovirus-like particles (NoV-LPs) [48]. As the previous study, the fluorescence enhancement of QDs was triggered by the steric hindrance-induced LSPR signal from the adjacent AuNPs. The linear response to the concentration of NoV-LPs was in the 10^−14^ to 10^−9^ g mL^−1^ range, showing a limit of detection of 12.1 × 10^−15^ g mL^−1^. The selectivity for the detection of the target NoV-LPs was evaluated with influenza virus A (H3N2) and Zika viruses, demonstrating the nanobiosensor specificity (Figure 8).

**Figure 9:**
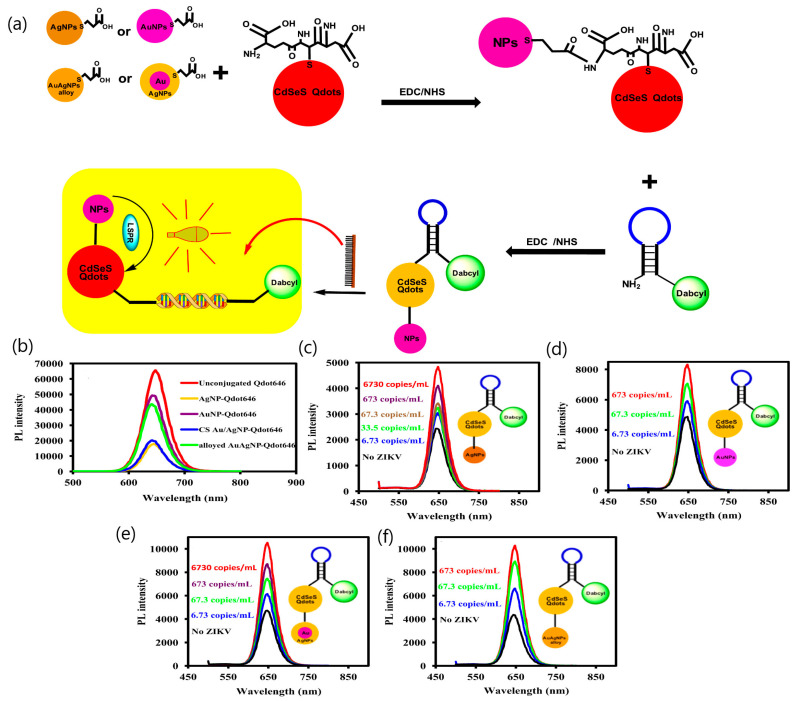
(**a**) Illustration of the conjugation of NPs to GSH (glutathione)-CdSeS QDs (Qdot646) to form the plasmonic NP-QDs’ nanohybrids. The subsequent conjugation of the plasmonic NP-QDs to the MB to form the plasmonic NP-QDs-MB biosensor probe and the LSPR-mediated fluorescence signal detection of the Zika virus based on hybridization of the RNA with the DNA loop sequence of the MB. (**b**) Fluorescence-quenching effect of the plasmonic NPs on the fluorescence of Qdot646 particles. (**c**–**f**) LSPR-mediated fluorescence enhancement of Zika virus RNA using the plasmonic NP-QDs-MB biosensor probe: (**c**) AgNP-Qdot646-MB, (**d**) AuNP-Qdot646-MB, (**e**) CS Au/AgNP-Qdot646-MB (molecular beacon), and (**f**) alloyed AuAgNP-Qdot646-MB. Inset: Structure of the plasmonic NP-QD-MB biosensor probe. The fluorescence measurement was carried out in the wavelength range of 480–800 nm with an excitation wavelength of 470 nm. The hybridization time was set at 3 minutes. Adapted with permission from Adegoke et al. [49] Copyright © (2017) Elsevier.

Finally, another approach consisting of four different plasmonic NPs bound to CdSeS alloyed QDs was developed to determine Zika RNA virus [49]. The investigation of all four fluorescent nanohybrids’ systems demonstrated that the LSPR fluorescence signal is strongly dependent on the plasmonic nanostructure, showing higher intensity for the bimetallic NPs in comparison with the single-metallic plasmonic NPs. The limits of detection of the nanohybrids were between 1.7 and 7.6 copies mL^−1^, depending on the composition of the nanohybrids: Alloyed AuAgNP-Qdot646-MB (molecular beacon) (1.7 copies mL^−1^) > CS Au/AgNP-Qdot646-MB (LOD = 2.4 copies mL^−1^) > AuNP-Qdot646-MB (LOD = 2.9 copies mL^−1^) > AgNP-Qdot646-MB (LOD = 7.6 copies mL^−1^) (Figure 9). The work proved that LSPR signals from plasmonic nanoparticles (NPs) can modulate the fluorescence signal from semiconductor QDs.

#### 2.2.2. Carbon-Based Nanoparticles

Carbon nanomaterials are graphite structures that can adopt different dimensions ranging from cylindrical nanotubes to plane nanosheets. Graphene-based nanostructures provide numerous benefits for plasmonic applications due to their physicochemical properties including a high surface and biocompatible area that facilitate surface functionalization [60,61].

**Figure 10:**
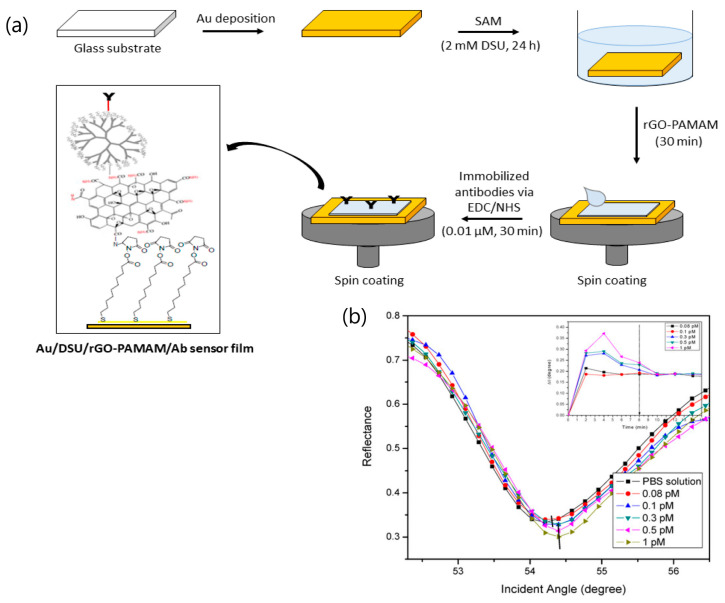
(**a**) Preparation procedure of the sensor film. SAM: Self-assembled monolayer, EDC:N-ethyl-N-(3-(dimethylaminopropyl) carbodiimide, NHS: N-hydroxysuccinimide, DSU/rGO–PAMAM: dithiobis (succinimidyl undecanoate)/reduce graphene oxide–polyamidoamine; (**b**) SPR reflectance of the SPR sensor based on a DSU/amine-functionalized rGO–PAMAM/IgM sensor film. Inset: SPR time response upon introduction of 0.08 to 1 pM of dengue virus (DENV 2) E-proteins. Adapted with permission from Omar et al. [50] Copyright © (2020) MDPI (under the terms and conditions of the Creative Commons CC BY License).

Several SPR graphene-based applications have been developed by Mahdi’s group for the quantification of dengue virus at picomolar levels. A composite of reduced graphene oxide and polyamidoamine (PAMAM) dendrimers is described for the detection of the E-proteins of the serotype 2 of dengue virus (DENV 2). The detection format consisted of an immunoassay in which monoclonal antibodies were immobilized on self-assembled, dithiobis (succinimidyl undecanoate) amine-activated layers [50]. The characterization of analytical parameters involved the determination of affinity-binding constants, detection accuracy, and figure of merit values for the minimum E-proteins’ concentration (0.08 pM) while the assay selectivity was tested against E- proteins of Zika virus (Figure 10). A similar SPR immunosensor strategy, although involving the introduction of cadmium sulfide quantum dots over graphene oxide surfaces, was also applied to the detection of dengue E-proteins’ virus [51]. The optimization of protein interactions with the antibody immobilized on the graphene oxide-activated surface achieved the lowest detection limit at 1 pM, thus demonstrating less sensitivity in comparison with the composite of PAMAM dendrimers.

Other plasmonic immunosensing approaches have exploited the potential of binary nanoparticle-graphene hybrid structures for the detection of virus in biological samples. For example, a magnetofluorometric platform made use of the combined effect of SERS and fluorescence of quantum dots to assist nanoparticle-decorated carbon graphenes in monitoring the presence of hemagglutinin (HA) protein in the surface of H1N1 influenza virus [52]. The immunoassay involved a sandwich structure between binary nanoparticles-graphene hybrids and QDs through HA Ab-virus conjugation targeting the virus by magnetic separation and subsequent fluorometric detection. The influence of complex media in the assay performance was tested using spiked serum samples achieving a LOD of 6.07 pg mL^−1^. Likewise, the same sensing scheme involving binary nanoparticle-graphene structures, although with the combined effect of gold and magnetic nanoparticles, was presented to detect norovirus-like particles [53]. In this case, the fluorescent signal enhancement provided by QDs was replaced by the electrical conductivity and magnetic properties of the metallic and magnetic nanoparticles decorated with graphene. The change in electrical resistance was used to measure the capture of the norovirus-like particles with a detection limit calculated as 1.16 pg mL^−1^.

Finally, another composite consisting of gold nanorods and graphene oxide and gold nanorods (GO-GNRs) was applied to the determination of hepatitis B surface antigen using surface-enhanced Raman spectroscopy (SERS) as described above [45].

## 3. Other Plasmonic Nanomaterial-Based Strategies

### 3.1. Nanopatterning, Nanostructures

The fabrication of plasmonic structures also relies on lithographic approaches, which enable the design of ordered arrays of metallic nanoapertures, such as nanoholes, nanoantennas, nanoslits, or nanodisks. The interaction of light with periodic arrays of nanoholes exhibits extraordinary optical transmission (EOT) effects, thus enhancing the transmission efficiency of light at certain wavelengths. These spectral features have facilitated the development of optical arrangements that support high bulk sensitivities and permit their integration into microfluidic systems and their application for the detection of single molecules [62,63,64,65].

**Figure 11:**
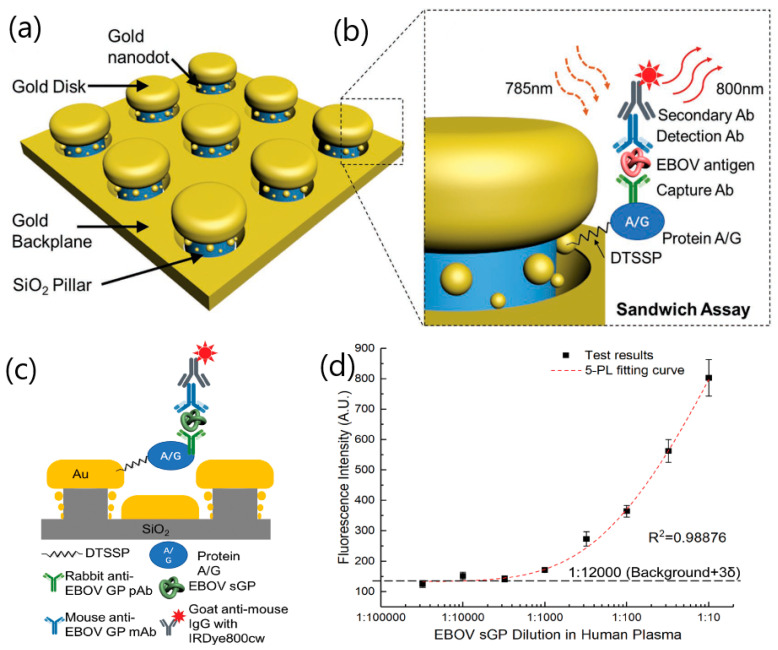
The Ebola virus sensor nanostructures and fluorescence sensing methods. (**a**) Schematic of nanoantenna array. (**b**) Exploded view showing the details of single nanopillar structures and a sample Ebola virus sandwich assay structure on chip. Ebola virus soluble glycoprotein (EBOV-sGP))-spiked human plasma study using the nanoantenna array biosensor. (**c**) EBOV sGP sandwich assay format. (**d**) Calibration curve of sGP tested on the nanoantenna array (N = 27, error bars stand for standard deviations). The nanoantenna array nanostructures absorb light at the resonance wavelength of 700 nm with over 95% light absorption efficiency. The resonance peak can blue-shift for over 50 nm in wavelength through increasing the SiO2 pillar height from 52 to 61 nm. Adapted with permission from Zang et al. [54] Copyright © (2020) Wiley.

For instance, Zang et al. presented an interesting approach for single-molecule detection of Ebola virus antigens. The biosensor consisted of a 3D plasmonic nanoantenna array, which used a sandwich-immunoassay format with fluorescent intensity enhancement [54] (Figure 11). The nanoantenna configuration involved silicon-dioxide nanopillars paved with gold nanodisks and nanodots that enhanced the fluorescence signal through the formation of nanocavities. The functionalization of the nanopillars’ surface using a thiol–gold link and a protein A/G layer as spacers allowed the selection of the best capture and detection antibody while preventing irradiative fluorescence signal loss on the gold surfaces. Under optimized conditions, the Ebola virus soluble glycoprotein (EBOV-sGP) was detected in human plasma at 220 fg mL^−1^ levels, which significantly improved the recommended immunoassay-best test for the Ebola virus antigen detection.

Another plasmonic nanohole-based assay was developed for capturing single dengue virus-like particles [55]. The dimensions of the nanohole array platform were calculated according to the size distribution of virus particles toward a virucidal drug candidate by nanoparticle tracking analysis. The work specifically concentrated on the investigation of the SPR response to the adsorption of virus-like particles into either nonfunctionalized or thiol-terminated, methoxypolyethylene(glycol)-functionalized nanohole arrays. SPR experiments indicated higher bulk refractive index sensitivities in functionalized nanoholes with a low surface coverage with regard to nonfunctionalized nanoholes. The biosensing performance was also evaluated by monitoring spectral shifts for the virucidal-induced rupture of single virus-like particles, thereby showing that almost all virus-like particles were ruptured on the mPEG (methoxy polyethylene glycol)-SH-functionalized nanohole array.

Similarly, another study measured the adsorption of a small virus particle of 25 nm (PhiX174) using double nanohole apertures in a gold film by the extraordinary acoustic Raman (EAR) method [56]. As with the previous work, the research concentrated on the characterization of the optical trapping and analytical results on virus sensitivity and selectivity were not evaluated.

### 3.2. Fiber Optic

SPR and LSPR biosensors that integrate optical fibers for coupling optical excitation of surface plasmons offer an interesting alternative to classical prism-based configurations. The possibility of driving the propagation of the electromagnetic field radiation through a fiber-optic system permits the improvement of the wavelength modulation and the development of compact structures. Many optical fiber SPR/LSPR platforms have provided miniaturized sensing approaches for the determination of clinical biomarkers [32]. Most representative advancements in fiber-optic strategies for viral diagnosis are presented in Table 2.

In particular, a fiber-optic SPR system has been described for the analysis of avian influenza virus subtype H6 [66]. To optimize the immobilization of the self-assembled monolayers (SAM) and the subsequent antibody functionalization, the optical fiber was modified with plasma at low temperature, which rendered better results than chemical modification. The binding interaction between immobilized antibodies and antigens on the cell surface was evaluated in the 10^4^ to 10^8^ EID 50/0.1 mL (EID: embryo-infectious dose), showing a detection limit of 5.14 × 10^5^ EID 50/0.1 mL.

The combination of the optical properties of gold nanostructures-LSPR surfaces with the achievement of total internal reflection via optical fiber configurations can provide signal enhancement and better spatial sensitivities. The integration of nanorod-modified surfaces into a fiber-optic LSPR platform have permitted the development of an immunosensor for the determination of a nonclinical application in plant viruses: Cymbidium mosaic virus (CymMV) and Odontoglossum ringspotvirus (ORSV) [67]. The limit of detection of viral antigens over antibody functionalized on gold nanorods was at picogram-per-liter levels (48–42). The improvement in sensitivity in comparison with ELISA method was attributed to the higher sensitivity of nanorods, which additionally prevents the color interference of similar-sized nanospheres.

Finally, the coating of an excessively tilted fiber grating surface (Ex-TFG) with gold nanospheres has permitted the development of an immunosensor for detecting Newcastle disease virus (NDV) [68]. The modification of the fiber cladding with gold nanospheres (AuNs) enhanced the LSPR effect while the activation of gold nanospheres with staphylococcal protein A (SPA) improved the bioactivity of anti-NDV monoclonal antibodies 5–10 times compared to that of reference Ex-TFG without AuN treatment. The monitoring of resonance wavelength red-shift showed a minimum detectable amount for NDV of ~5 pg, which was slightly better than RT-PCR (10 pg) method. The specificity of the immunoassay was also demonstrated against avian influenza virus while clinical performance confirmed by comparing results of NVD in allantoic fluid and solution test.

### 3.3. Colorimetric

The utilization of colorimetric assays is becoming more important in clinical diagnosis for detection of either organic (proteins, DNA) or inorganic molecules (anions, metal ions). The fabrication of plasmonic colorimetric nanosensors relies mainly on noble metal nanoparticles due to their high LSPR extinction coefficients in the visible range [27,30]. Most of the plasmonic colorimetric assays make use of the aggregation of metallic nanoparticles, resulting in a shift of the plasmonic band from red to blue that can be easily observed by naked eyes [77]. The distance between nanoparticles is responsible for the red shift in the plasmon bands when the interparticle distance decreases to less than the average particle diameter. In addition to the aggregation effect, the LSPR shift can also be induced by the transformation of the size, morphology, and dielectric environment, as a consequence of the etching or the growth of metal plasmonic nanoparticles [77]. Although the strong plasmonic coupling between nearby particles is the most exploited approach, “non-aggregation” plasmonic colorimetric sensors are attracting attention due to their simplicity and easy use as test strips.

A plasmonic colorimetric sensor based on the aggregation of colloidal nanoparticles was described for the detection of influenza virus [69]. The application utilizes the interaction between viral envelope protein hemagglutinin and sialic acid to reduce and stabilize gold nanoparticles’ aggregation. Although sensitivity values related to virus quantification were not shown, a good correlation between variations in optical density and influenza B virus dilutions, causing a colorimetric change, was reported for 0.156 vol% virus dilutions (hemagglutination titer of 512).

Another plasmonic colorimetric application for influenza virus detection (lineage H5N1) employed gold nanobipyramids (Au NBPs) as noble metal nanostructures [70]. This approach proved the deposition of silver on highly monodispersed gold nanobipyramids after the reaction catalyzed by alkaline phosphatase (ALP). The silver coating on the Au NBPs caused a blue shift in the LSPR band, resulting in a vivid color change that could be detected with the naked eye. The proposed method was applied to the determination of ALP activity showing a limit of detection of 0.086 mU mL^−1^ while H5N1 virus antigen was detected at 1 pg mL^−1^ levels using a sandwich-immunoassay format. The improvement in sensitivity in comparison with other methods was attributed to the high index faceted on the tips of the gold nanobipyramids with regard to gold nanorods.

**Figure 12:**
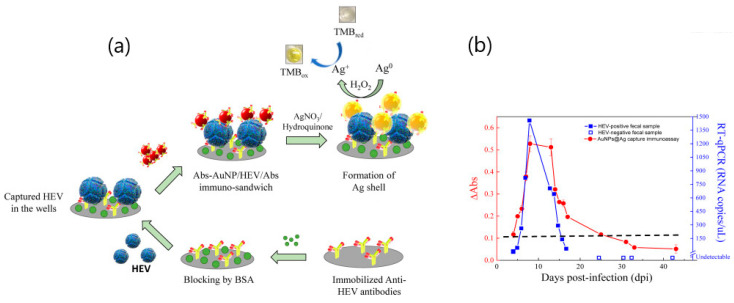
(**a**) Schematic principle of the silver-deposited, gold nanozyme-based capture immunoassay representing the binding of Ab(antibody)-AuNPs on the captured HEV-LPs (hepatitis E virus-like particles), Ag-shell deposition on the on the surface of AuNPs, and color development by the catalytic oxidation of tetramethylbenzidine (TMBZ) by Ag^+^ and H_2_O_2_. (**b**) Comparison of the HEV detection sensitivity between the proposed AuNPs@Ag capture immunoassay (closed circles) and RT-qPCR (closed squares) in fecal specimens (error bars represents the standard deviation of triple measurements). Open squares represent the viral RNAs, which are undetectable in RT-qPCR. Surface of AuNPs. The developed color was measured at 450 nm with reference wavelength of 655 nm. Adapted with permission from Khoris et al. [71] Copyright © (2020) Elsevier.

Similarly, a plasmonic platform consisting of silver deposition on antibody-conjugated gold nanoparticles was applied for monitoring hepatitis E virus (HEV) [71]. A nanozyme-based immunoassay was used to capture HEV-like particles (HEV-LPs) via binding to the conjugated gold nanoparticles (Figure 12). Color development was obtained by enhancing the catalytic activity of silver by adding tetramethylbenzidine (TMBZ) and hydrogen peroxide (H_2_O_2_). A limit of detection of 10 pg mL^−1^ was obtained in buffer solution while the determination of HEV in real samples collected from fecal matter of HEV-infected monkey was in good correlation with RT-qPCR conventional methods (Figure).

Finally, a colorimetric assay approach based on the LSPR changes of silver nanoparticles induced by the hybridization chain reaction of the microRNA biomarker (miR-29a-3p) was described to detect H1N1 influenza A virus at picomolar levels [72]. The hybridization chain reaction triggered by target microRNA generated changes in the DNA stability that affected the silver nanoparticles’ colloid system and was reflected by UV-visible spectrum. The limit of detection achieved by the proposed method was comparable to other colorimetric assays.

### 3.4. Electrochemiluminescence

The effect of collective oscillations of free electrons on the surface of metal nanostructures displayed in LSPR sensing can enhance the optical signal of a luminophore near noble metal nanoparticles [31,73]. Plasmonic-enhanced electrochemiluminescence can be explained by the increase of excitation and emission, resulting from the enhancement of the electromagnetic field of incident light [31]. The distance between plasmonic nanoparticles and the luminophore determines the electrochemiluminescence process since the signal could be quenched when plasmonic nanoparticles working as energy or electron acceptors are very close to the luminophore. As a result, the energy transfer can be controlled using DNA or adaptors for the distance-mediated quenching and enhancement of electrochemiluminescence signal [74]. Owing to the simplicity of optical configuration, high sensitivity, and low background noise, plasmon-enhanced electrochemiluminescence has emerged as a promising biosensing scheme for the fabrication of medical diagnostic applications [74].

**Figure 13:**
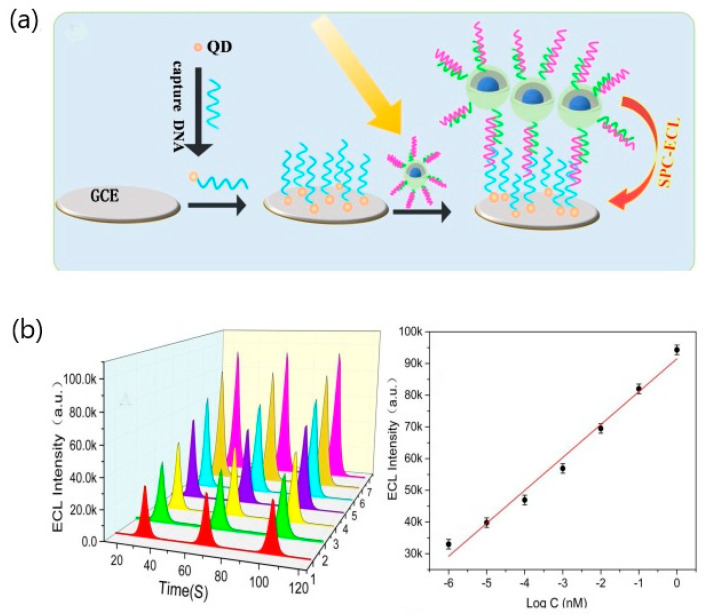
(**a**) The preparation process of magnetic-plasmonic Fe_3_O_4_ yolk/Au shell (M@Au). (**b**) Schematic illustration of plasmon-enhanced, electrochemiluminescence (ECL)-sensing process (glassy carbon electrode (GCE), surface plasmon coupling (SPC)). The plasmon resonance peak of M@Au first red shifted from 521 nm to 548 nm and blue shifted from 548 nm to 531 nm. Adapted with permission from Zhang et al. [73] Copyright © (2020) Elsevier.

Although the number of virus-sensing approaches is still limited, several works have explored the potential of plasmon-enhanced electrochemiluminescence (ECL) as clinical nanosensors. For example, an ECL assay based on magnetic-plasmonic Fe_3_O_4_ yolk/Au shell (M@Au) was reported for the detection of a mouse sarcoma virus oncogene [73]. The study exploited the advantages of combining the magnetic properties of internal Fe_3_O_4_ nanoparticles and the stability and low toxicity of graphite quantum dots to detect nucleic acids in the 1-fM−1-nM range (Figure 13). The effect of the outer gold shell demonstrated better surface coupling than conventional gold nanoparticles. Particularly, the surface coupling induced by M@Au caused the amplification of the quantum dots signal due to the hybridization between target DNA and capture DNA labeled to M@Au. Practical application in spiked serum samples showed recoveries from 90.0–103%, demonstrating its usefulness for clinical analysis.

Another plasmon-based ECL strategy utilizes the strong surface plasmon-coupling nonmetallic MoS_2_ nanosheets to enhance the ECL signal of sulfur-doped boron nitrogen QDs (S-BN QDs) [74]. The application investigated the effect of the distance between the luminophore and the nanoparticles using DNA chains of different lengths. The increase of distance between MoS_2_ nanosheets and S-BN QDs reduced the energy transfer while strengthening the surface plasma-coupling effect. Specifically, the assay was applied to the detection of hepatitis C virus (HCV) gene. The so-called hybridization chain reaction was used as an isothermal enzyme-free DNA amplification method to quantify DNA HCV with a limit of detection (LOD) of 0.17 pmol L^−1^.

## 4. Plasmonic Advancements in COVID-19 Diagnosis

**Figure 14:**
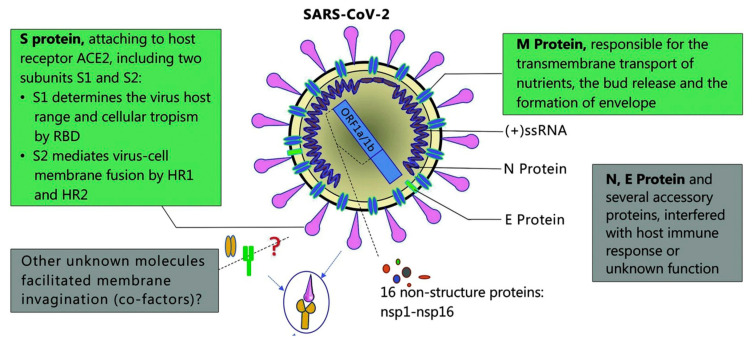
Schematic diagram of the Severe acute respiratory syndrome coronavirus 2 (SARS-CoV-2) structure. Structural proteins, including spike (S) glycoprotein, small envelope (E) protein, matrix (M) protein, and nucleocapsid (N) protein, and also several accessory proteins. Adapted with permission from Guo et al. [78] Copyright © (2020) BMC Springer Nature.

The SARS-CoV-2 is an enveloped, nonsegmented β-coronavirus that causes a new, severe, acute respiratory syndrome and coronavirus disease (COVID-19) [1,3,75,78]. Since it is a positive-sense, single-stranded RNA virus (~30 kilobases in size and ~9860 amino acids), the genome of SARS-CoV-2 is the most important biomarker for diagnosis of COVID-19. SARS-CoV-2 structural and accessory proteins and even the whole virus SARS-CoV-2 could be used as antigens to monitor coronavirus disease (see Figure 14). Additionally, the detection of immunoglobulin M (IgM) and immunoglobulin G (IgG) responses after five days of the disease onset can also be utilized as indicators of COVID-19. As a result, reverse-transcription polymerase chain reaction (RT-PCR), gene sequencing, ELISA, and lateral flow immunoassay are the main diagnostic techniques at the moment [4,76].

**Figure 15:**
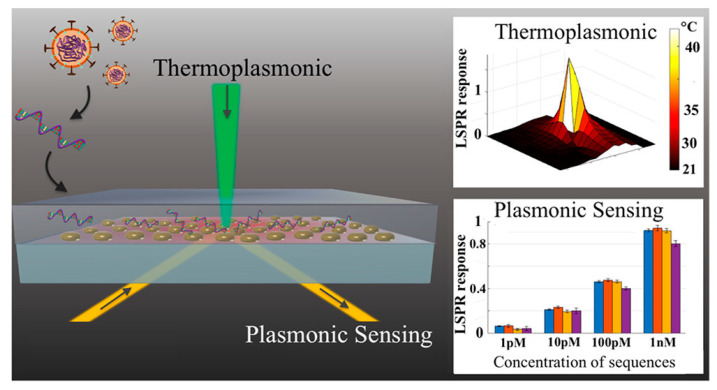
Schematic representation of two-dimensional gold nanoislands functionalized with complementary DNA receptors, mapping the temperature distribution around the plasmonic photothermal (PPT) heat source and concentrations of various viral oligos measured using the dual-functional LSPR biosensors. An excitation laser with 532-nm peak wavelength and 40-mW maximum optical power was applied onto the gold nanoislands (AuNI) chip in the normal incident angle to improve the conversion efficiency of thermoplasmonic. Adapted with permission from Qiu et al. [75] Copyright © (2020) American Chemical Society.

Nevertheless, the search for rapid and reliable point-of-care devices has triggered the development of plasmonic methods based on the latest technological advancements. For example, a novel approach combines the plasmonic photothermal (PPT) effect and LSPR sensing to detect DNA-selected sequences via hybridization to DNA receptors immobilized on two-dimensional gold nanoislands (AuNIs) (Figure 15). This dual-functional plasmonic biosensor takes advantage of the PPT heat generated on the AuNIs’ chip to increase the hybridization temperature and discriminate two similar gene sequences (RdRp genes) from SARS-CoV and SARS-CoV-2. A detection limit of 0.22 pM was obtained using a multigene mixture including the DNA sequences RdRp-COVID, open reading frame 1ab (ORF1ab)-COVID, and E genes from SARS-Cov-2. The thermoplasmonic enhancement also contributed to improve the stability of the assay by using two different angles of incidence to excite the plasmonic resonances of PPT and LSPR at two different wavelengths. Since the proposed method utilizes the same criteria for hybridization of nucleic acids, based on the decrease of the melting temperature than PCR-test, the potential to complement this technique is suggested.

Another innovative approach for COVID-19 diagnosis comprised the development of a colorimetric assay based on gold nanoparticles (AuNPs) functionalized with thiol-modified antisense oligonucleotides (ASOs) specific for N-gene (nucleocapsid phosphoprotein) of SARS-CoV-2 [76]. The biosensing scheme comprised the change in its SPR absorbance spectra with a red shift of ~40 nm when thiol-modified AuNPs agglomerated selectively in the presence of their target RNA sequence. This application also demonstrated that the addition of endonuclease Ribonuclease (RNAse H) leads to a visually detectable colorimetric change due to the agglomeration among the AuNPs, resulting from the cleaving of the RNA strand from the composite hybrid of RNA and Au-ASO composite. The assay selectivity was measured in the presence of MERS-CoV viral RNA, showing a limit of detection of 0.18 ng μL^−1^ of RNA with SARS-CoV-2 viral load. The main advantage of the proposed method was the possibility of being applied to target other regions of the viral genomic material, such as S-gene (surface glycoprotein), E-gene (envelope protein), and M-gene (membrane glycoprotein) without using sophisticated instrumental techniques.

## 5. Conclusions and Future Perspectives

The uncertain proportions of pandemic outbreaks have triggered the need for reliable and cost-effective protocols easily adaptable to the changing virulence of virus strains. In recent years, plasmonic biosensors are being increasingly applied for clinical diagnosis of viral and other infectious diseases. Typical plasmonic biosensing strategies rely on the versatility of SPR and LSPR as label-free detection systems capable of monitoring binding interactions in a short period of time. Nevertheless, the incorporation of technological advancements has precipitated the development of nanomaterial-based applications for improving the sensitivity and specificity of classical configurations. The unique optical properties of plasmonic nanostructures has been exploited in combination with SERS colorimetric, fluorescence, or luminescence enhancement for viral diagnosis. Likewise, the development of plasmonic virus-sensing approaches has also benefitted from the variety of virus biomarkers. Thus, a high number of virus plasmonic biosensors have prompted the advance of novel functionalization strategies to achieve the effective coverage of the biological receptor while ensuring the affinity and specificity towards the target viral nucleic acids, proteins, or whole virus.

Despite this substantial progress, current plasmonic applications have not yet outpaced conventional laboratory-based sequencing and immunoanalytical techniques. The improvement of sensing schemes has led to the attainment of ultrasensitive, robust, and reproducible analytical performances. However, the automation and integration of microfluidics as well as the prevention of nonspecific proteins’ adhesion in complex media still remains a challenge in many cases. On the one hand, the fabrication of automatable point-of-care devices is strongly dependent on both the design of reliable microfluidics and the integration of advanced software in miniaturized platforms. In this sense, the utilization of novel flexible materials via nanopatterning lithographic approaches may facilitate the incorporation of microchannels that enable the delivery of microvolume samples without requiring additional equipment. Secondly, plasmonic virus detection from clinical specimens is still limited due to the lack of universal methods that prevent the interference of biomolecules present in undiluted body fluids. From this perspective, the design of effective antifouling coatings, which take into account either the composition of the media or the biological receptor’s characteristics, will contribute to fill the gap between traditional analytical methods and plasmonics’ applications. By addressing these challenges, the huge potential for single virus detection along with the effectiveness and simplicity of current plasmonic configurations will impact on the routine surveillance of virus in clinical settings during this decade.

## Figures and Tables

**Table 1:** Key analytical features of nanomaterials-based sensors classified according to the characteristics of target virus, detection format (namely immobilization strategy or biological receptor), and limit of detection.

Nanomaterial: Plasmonic Configuration	Target Virus	Detection Format	LOD (Media/Biological Sample)	Reference
Metal nanoparticles				
LSPR	Hepatitis B surface antigen	Hetero-assembled AuNPs sandwich-immunoassay	100 fg mL^−1^ (human serum spiked samples)	[40]
	Avian influenza virus (AIV H5N1) hemagglutinin protein	Au spike-like nanoparticle onto indium-tin-oxide functionalized with DNA	63.4 pg mL^−1^ (10% diluted chicken serum) ^1^	[41]
	Dengue NS1 antigen	Thin silver film onto silicon substrate-Immunoassay/ Polyethersulfone membrane filter	~0.06 μg mL^−1^ (Spiked whole blood samples)	[42]
	Respiratory Syncytial Virus	Antibody-functionalized gold, silver and copper nanoparticles/Intact virus	2.4 plaque forming units (PFU) (eagle’s minimum essential medium)	[43]
SERS	Viral DNA (M13mp18single-stranded DNA)	Nanoclusters of Au NP functionalized with Virus Like Particles	0.25 ng μL^−1^ (bulk solution: water)	[44]
	Hepatitis B surface antigen	Composite gold nanorods and graphene sandwich-immunoassay	0.05 pg mL^−1^ (9 patients diluted serum)	[45]
Quantum dots				
LSPR-induced fluorescence	Influenza virus H1N1 antigens	CdSeTeS QD functionalized with anti-neuraminidase antibody and AuNP conjugated to anti-hemagglutinin antibody-immunofluorescence assay	0.03 pg mL^−1^ (deionized water) and 0.4 pg mL^−1^ (human serum)	[46]
	Influenza virus H1N1 antigens	CdZnSeS/ZnSeS QDs and gold nanoparticles- controlled distance and fluorescence quenching- immunofluorescence assay	17.02 fg mL^−1^ (deionized water) and 65.1 fg mL^−1^ (10% diluted human serum)	[47]
	Norovirus	Composites of Cysteine capped CdSeTeS QDs and AuNPs- fluorescence quenching- immunofluorescence assay	12.1 × 10^−15^ g mL^−1^(10% diluted human serum)	[48]
	Zika RNA virus	Nanohybrids of NPs bound to CdSeS alloyed QDs-hybridization	2.4–7.6 copies mL^−1^ (assay buffer)	[49]
Carbon-based				
SPR	Dengue virus (E-proteins, serotype 2)	Composite of reduce graphene oxide and polyamidoamine (PAMAM) self-assembled to dithiobis (succinimidyl undecanoate) amine-activated layers- immunoassay antibody immobilization	4.24 pg mL^−1^ (PBS solution) ^1^	[50]
	Dengue virus (E-proteins, serotype 2)	Cadmium sulfide quantum dots over graphene oxide	53 pg mL^−1^ (PBS solution) ^1^	[51]
SERS magneto fluorometric	Influenza virus H1N1 hemagglutinin protein	Binary AuNP-graphene hybrids and QD through antibody conjugated immunoassay a sandwich structure	7.02 fg mL^−1^ (deionized water) 6.07 pg mL^−1^ (human serum)	[52]
	Norovirus like particles	Binary Metallic and magnetic nanoparticles decorated with graphene-immunoassay	1.16 pg mL^−1^ (2% BSA solution)	[53]
Nanopatterning nanostructures				
Plasmonic fluorescence	Ebola virus soluble glycoprotein	3D plasmonic nanoantenna array Sandwich immunoassay format with fluorescent intensity enhancement	220 fg mL^−1^ (diluted human plasma)	[54]
SPR	Dengue virus like particles	mPEG (polyethylene glycol)-SH-functionalized nanohole array.	Not stated	[55]
EAR (extraordinary acoustic Raman)	Virus particle of 25 nm (PhiX174)	Optical trapping	Not stated	[56]

^1^ Values recalculated to express in mL; LOD: Limit of detection; AuNPs: Gold nanoparticles.

**Table 2:** Key analytical features of other biosensing strategies classified according to the characteristics of target virus, detection format (namely immobilization strategy or biological receptor), and limit of detection.

Plasmonic Configuration	Target Virus	Detection Format	LOD (Media/Biological Sample)	Reference
Fiber optic				
SPR	Avian influenza virus subtype H6 antigens	Cold plasma optical fiber modification Immunoassay: antibody immobilization	5.14 × 10^5^ EID 50/0.1 mL (assay buffer)	[66]
LSPR	Cymbidium mosaic virus /Odontoglossum ringspotvirus	Gold nanorods Immunoassay: antibody functionalization	48–42 pg L^−1^ (saps)	[67]
LSPR Fiber Grating	Newcastle disease virus	Modification of the fiber cladding with gold nanospheres (AuNs) activated with staphylococcal protein A–Immunoassay	~5 pg (allantoic fluid and buffer)	[68]
Colorimetric				
LSPR colorimetric	Influenza B virus	Stabilization of gold nanoparticle aggregation via the interaction of protein hemagglutinin and sialic acid	Not stated	[69]
	Influenza virus detection (H5N1 antigen)	Deposition of silver on highly monodispersed gold nanobipyramids-sandwich immunoassay format	1 pg mL^−1^ (human serum samples)	[70]
	Hepatitis E virus antigen	Silver deposition on antibody conjugated gold nanoparticles-nanozyme-based immunoassay	10 pg mL^−1^ (fecal matter)	[71]
	Influenza A virus H1N1	Hybridization chain reaction of the microRNA biomarker (miR-29a-3p)	1 pM (assay buffer)	[72]
Electrochemiluminescence				
Magnetic	Sarcoma virus oncogene (DNA virus)	Combination of magnetic-plasmonic Fe_3_O_4_ yolk/Au shell and graphite nitride quantum dots	0.3 fM (human serum)	[73]
Plasmon-based	Hepatitis C virus (DNA virus)	MoS_2_ nanosheets combined with sulfur doped boron nitrogen QDs -hybridization reaction chain	0.17 pM	[74]
COVID-19 Plasmonic advancements				
LSPR Photothermal	SARS-CoV-2 (DNA sequences)	Gold nanoislands to increase the hybridization temperature and discriminate two similar gene sequences	0.22 pM	[75]
Colorimetric	SARS-CoV-2 (RNA sequence)	Gold nanoparticles functionalized with thiol modified antisense oligonucleotides specific for N-gene (nucleocapsid phosphoprotein)	0.18 ng μL^−1^	[76]

LOD: Limit of detection; AuNPs: Gold nanoparticles.
